# Does the Rarity of a Flower’s Scent Phenotype in a Deceptive Orchid Explain Its Pollination Success?

**DOI:** 10.3389/fpls.2020.584081

**Published:** 2020-12-16

**Authors:** Herbert Braunschmid, Stefan Dötterl

**Affiliations:** Department of Biosciences, Plant Ecology, Paris-Lodron-University of Salzburg, Salzburg, Austria

**Keywords:** evolution, floral volatiles, orchids, variability, frequency-dependent selection, pollinator-mediated selection, *Cypripedium calceolus*, quantitative traits

## Abstract

Floral scent, a key mediator in plant–pollinator interactions, varies not only among plant species, but also within species. In deceptive plants, it is assumed that variation in floral scents and other traits involved in pollinator attraction is maintained by negative frequency-dependent selection, i.e., rare phenotypes are more attractive to pollinators and hence, have a higher fitness than common phenotypes. So far, it is unknown whether the rarity of multivariate and/or continuous floral scent traits influences the pollination success of flowers. Here, we tested in the deceptive orchid *Cypripedium calceolus*, whether flowers with rarer scent bouquets within a population have a higher chance to getting pollinated than flowers with more common scents. We collected the scent of more than 100 flowers in two populations by dynamic headspace and analyzed the samples by gas chromatography coupled to mass spectrometry (GC/MS). From the same flowers we also recorded whether they set a fruit or not. We introduced rarity measures of uni- and multivariate floral scent traits for single flowers, which allowed us to finally test for frequency-dependent pollination, a prerequisite for negative frequency-dependent selection. Our results do not show rarity has an effect on the likelihood to set fruits in neither of the two populations and in none of the scent characteristics analyzed. Hence, there is no evidence of negative frequency-dependent pollination mediated by the floral scent of *C. calceolus*. We discuss that our approach to determine rarity of a scent is applicable to any univariate or multivariate (semi)quantitative trait.

## Introduction

Most angiosperm species are pollinated by animals, mainly insects (Ollerton et al., 2011), and flowers of animal-pollinated plants typically advertise their presence by visual and olfactory cues (Chittka and Raine, 2006). These cues vary among and within species (Schiestl, 2005). The variation in floral traits is believed to be especially high in deceptive plants, which signal the presence of a reward without providing it (e.g., Dafni, 1984; Renner, 2006). This is because the variability in such signaling traits increases the difficulty for pollinators to recognize and learn to avoid cheating plants (Schiestl, 2005). Hence, it is expected that common phenotypes have a lower reproductive success than rare phenotypes. The evolutionary process by which the fitness of a phenotype depends on its frequency relative to other phenotypes in a population, is called frequency-dependent selection (Futuyma and Kirkpatrick, 2017). In rewarding plants, positive frequency-dependent selection is expected, in deceptive plants, negative frequency-dependent selection.

Negative frequency-dependent selection has been studied in many groups of living beings (reviews in Ayala and Campbel, 1974; Delph and Kelly, 2014; Brisson, 2018). For plants, these include studies of self-incompatibility, heterostyly, host-pathogen systems, and flower color. So far, negative frequency-dependent selection exerted by pollinators has mostly been investigated in plants that are dimorphic in flower color. The bumblebee-pollinated orchid *Dactylorhiza sambucina* maintains a color dimorphism of yellow and purple flowers with the reproductive success being negatively correlated with relative morph frequencies in an experimental setting (Gigord et al., 2001). Subsequent studies have compared morph frequencies with reproductive success, but hardly found any correlation (Pellegrino et al., 2005; Jersáková et al., 2006). Also, preferred switching of pollinators to rare phenotypes in natural populations could not be confirmed (Groiß et al., 2017), as Smithson and Macnair (1997) observed in a study with artificial flowers. Studies in two other color dimorphic deceptive orchid species did not show evidence of frequency-dependent selection either (Aragón and Ackerman, 2004; Ackerman and Carromero, 2005). One study on pollinator-mediated frequency-dependent selection investigated the deceptive orchid *Psychilis monensis*, which shows a continuous variation in flower color (Aragón and Ackerman, 2004). However, the authors reduced the variation by painting the labellum to obtain three uniform flower color categories. They also did not find significant effects. Hence, the study of Gigord et al. (2001) stands out as the only one showing negative frequency-dependent selection on floral colors. They worked with artificial plots at sites that were similar to natural habitats of *D. sambucina*. Though likely present, the authors did not record co-flowering species that might have influenced the behavior of pollinators. Thus, negative frequency-dependent selection was detected despite other processes were potentially active, such as facilitation (Peter and Johnson, 2008) and competition (Jersáková et al., 2006), that might have overwritten negative frequency-dependent selection in other studies.

The only study we know on frequency-dependent selection acting on floral scent was conducted with the epiphytic orchid *Tolumnia variegata*, a deceptive plant with two scent morphs: fragrant and odorless flowers (Ackerman et al., 1997). The authors built artificial plots with different frequencies of fragrant and odorless flowers and did not find a pattern of negative frequency-dependent selection. Flower scent is a complex communication medium between plants and their pollinators, and several qualitative and (semi-)quantitative continuous characteristics are relevant for pollinator attraction: the strength of the overall scent (total quantity), the presence or absence of individual components (qualitative composition), and the ratios of the scent components to each other (relative amounts; semi-quantitative composition) (reviewed in Raguso, 2008). The phenotype of a flower/inflorescence is univariate in its total quantity, but multivariate in its qualitative and semi-quantitative composition, because the scent of a single flower/inflorescence consists of up to several dozen components (e.g., 48 in this study; see section “Results”). Intraspecific differences in these traits determine the individuality of the scent of a flower or inflorescence.

A prerequisite for negative frequency-dependent selection acting on floral scent is that individuals with rare scent phenotypes in a population are more attractive to pollinators and have a higher reproductive success than individuals with more common phenotypes. So far, however, there is no study, which quantified the rarity of specific scent phenotypes within a population, and tested, whether the rarity of scent phenotypes correlates with the likelihood of a plant individual/flower to set fruit(s).

Here, we introduce rarity measures of floral scent for single flowers, and test whether rarity of a flower’s scent phenotype in the deceptive orchid *Cypripedium calceolus* L. explains its pollination success. Rarity was calculated for uni- and multivariate scent traits to be used for the tests on frequency-dependent pollination. *C. calceolus* is pollen limited, dependent on insects, mainly bees, for pollination, and emits a strong and variable scent (Nilsson, 1979; Bergström et al., 1992; Braunschmid et al., 2017). Various of its components are detectable by pollinating insects (Braunschmid et al., 2017). In this study, we collected floral scents in two natural populations by non-invasive dynamic headspace, and later in the season determined whether these same flowers set fruits. The scent was analyzed by gas chromatography and mass spectrometry. Based on pairwise resemblance measures of all different scent characteristics, chemical rarity values for all individual flowers were calculated. We then calculated logistic regressions to test for an effect of rarity in each floral scent characteristic on the likelihood to set fruits.

## Materials and Methods

### Plant Species

*Cypripedium calceolus* L. is a pollen-limited perennial orchid distributed in boreal and temperate forests of Europe and Asia (Cribb, 1997; Kull, 1999). It is one of the largest European orchids with a stem height of 20–60 cm, and a large and conspicuous flower with a labellum shaped like a shoe. The pollen consists of individual pollen grains aggregated in a sticky smear (Nilsson, 1979). The successful pollination of *C. calceolus* depends on insects, which are temporarily trapped in the labellum. They can only leave the slippery cave through a rear, narrow exit. Thereby, they first pass the stigma, where the pollen they might have collected from another flower is stripped off. Then, they squeeze past one of the two anthers, where pollen smear is loaded onto their backs (Nilsson, 1979, and references therein). This limits self-pollinations, although the plant is self-fertile. The plant grows vegetatively with horizontal rhizomes and forms patches with up to several dozen shoots.

### Study Sites

The investigations were carried out in the Austrian Limestone Alps near the city of Salzburg. Population Faistenau (FAI) is situated at 800 m above sea level on the eastern side of a mountain range in a forest characterized by *Picea abies* (L.) Karst., *Fagus sylvatica* L., and *Pinus mugo* Turra. Somewhat more than 300 shoots grow along a gravel stream within a 200 m radius. They are patchily distributed within this area. Groups consist of single plants and patches of up to 50 shoots. The Maria Alm population (MAA) is on the southern slope of a limestone mountain at 1,200 m above sea level, in an open mountain pine forest. More than 400 shoots are quite evenly distributed within a radius of approximately 300 m, but are still grouped in patches of up to 20 shoots. The flowering season of *C. calceolus* in the study populations was in May (FAI) and June (MAA). The populations flowered for about 3 weeks.

### Scent Collection and Analysis

Dynamic headspace samples of floral volatiles were collected *in situ* during daytime (10 a.m. to 3 p.m.) from individual flowers, using dynamic headspace methods (Dötterl et al., 2005). Flowers were enclosed in polyester oven bags and volatiles were trapped by pulling the air from the bag through small quartz adsorbent tubes (length: 15 mm, inner diameter: 2 mm; Hilgenberg GmbH, Malsfeld, Germany) for 8 min, using a rotary vane pump (G12/01 EB, Gardner Denver Thomas GmbH, Fürstenfeldbruck, Germany; flow rate: 200 ml/min). The tubes contained roughly 1.5 mg Tenax-TA (mesh 60–80) and 1.5 mg Carbotrap B (mesh 20–40; both Supelco) fixed by glass wool plugs (Heiduk et al., 2015). Samples collected from leaves and ambient air served as negative controls.

In population FAI the scents of 70 flowers available were sampled, of which 48 set a fruit and 22 did not set a fruit. In population MAA the scents of 210 flowers (all flowers available in sampling area) were sampled, of which only 18 set a fruit. Of the samples collected from flowers that did not set a fruit, we randomly selected a same number of flowers (18; Microsoft Excel, function RAND) to represent the frequency of phenotypes available among the non-pollinated flowers in the population. To verify that this sampling approach was representative, we performed two simulations (based on relative amounts of scent), one with samples of population FAI, and the second one with all samples included in the study (106; both populations, flowers with and without fruit set). In population FAI, we selected from the 48 flowers that set fruit 10,000 times 22 samples randomly, and every time tested whether rarity is a predictor of fruit set. In 99.5% of cases we found that rarity does not predict fruit set, which is in agreement with the analysis that included all data of population FAI (see section “Results”). A slightly different approach was taken for the simulations with all the 106 scent samples: out of these samples, we randomly took (again 10,000 times) 18 samples (as we did in the present study for population MAA) and calculated the mean rarity to see whether the mean rarity of the subsamples was the same as the mean rarity of the complete data set. Our simulations showed that 92% of our subsamples had the same mean rarity as the complete sample (single sample *t*-test *p* < 0.05).

The adsorbent tubes with the trapped volatiles were analyzed by gas chromatography coupled to mass spectrometry (GC/MS) using an automatic thermal desorption (TD) system (TD-20, Shimadzu, Japan) coupled to a Shimadzu GC/MS-QP2010 Ultra equipped with a ZB-5 fused silica column (5% phenyl polysiloxane; 60 m, i.d. 0.25 mm, film thickness 0.25 μm, Phenomenex), the same as used by Braunschmid et al. (2017). The samples were run with a split ratio of 1:1 and a consistent helium carrier gas flow of 1.5 ml/min. The GC oven temperature started at 40°C, then increased by 6°C/min to 250°C and was held for 1 min. The MS interface worked at 260°C. Mass spectra were taken at 70 eV (EI mode) from m/z 34 to 350. GC/MS data were processed using the GCMSsolution package, Version 4.11 (Shimadzu Corporation 1999–2013).

The compounds were (tentatively) identified with the databases ADAMS, ESSENTIALOILS-23P, FFNSC 2, and W9N11 as well as a database generated from synthetic standards available in the Plant Ecology Laboratory of the University of Salzburg. Total scent emission was estimated by injecting known amounts of monoterpenoids, aromatics, and aliphatics (Etl et al., 2016). Based on the compounds detected in *C. calceolus* (Braunschmid et al., 2017, present work), an own library was created and used for the semi-automatic analyses of samples. Compounds were included in the study if they were only present in flower samples or if the peak areas in flower samples were at least five times larger than in green leaf and ambient air controls.

To check whether the scent phenotype of single flowers is distinct, we sampled 12 flowers more than once (10 min to 5 days in between the replicate samples) and found that scent was indeed flower specific (ANOSIM: *R* = 0.80; *p* < 0.01). Thus, a single sample collected from a specific flower well characterizes the phenotype of this flower.

### Definition of Rarity

To measure how rare a scent phenotype is within a population, we use a formula that was originally developed by Violle et al. (2017) to define rarity in the context of biodiversity and ecosystem functioning. As a plant with a rare scent pattern is on average more dissimilar to the scent pattern of other plants within a population than a plant with a common scent pattern, pairwise dissimilarity/distance measures were used to learn about the rarity of and individual’s scent blend/characteristic. We determined the rarity of a specific scent sample as the mean of dissimilarities/distances of this sample with all other scent samples of a population. The higher the values in a specific data set, the rarer is a sample. Thus, we define rarity R_*i*_ of sample i within n samples as

R_*i*_ $=\frac{1}{n-1}\sum_{k=1;k\neq i}^{n}{\bar{D}}_{ik}$ with ${\bar{D}}_{ik}$ as dissimilarity or distance between *i* and *k*.

We used Euclidean distances for the univariate (total quantity), and Sørensen and Bray-Curtis dissimilarities for the multivariate qualitative (presence/absence of compounds) and semi-quantitative (percentage amounts of scent components within a flower in relation to the total amount) data sets, respectively. Bray-Curtis dissimilarities were calculated using the original percentage contribution of a single compound to the total scent, as well as on square-root and fourth-root transformed data to manipulate the importance of the main and minor compounds on the resemblance. These different scent characteristics were used for our analyses to meet possible olfactory search images of pollinators, which might be (more) based on total absolute amounts, qualitative, and/or semi-quantitative scent traits.

### Statistical Analysis

Similarities and dissimilarities in scent patterns among the samples were visualized using non-metric multidimensional scaling (NMDS), based on the Bray–Curtis dissimilarities calculated on untransformed relative amounts of compounds. A SIMPER analysis was used to determine the compounds most responsible for the similarity among samples. We used permutational multivariate ANOVA (PERMANOVA, 9,999 permutations, crossed design with fixed factors *fruit set* and *population*), to test for differences in scent among flowers which did or did not produce a fruit, also considering population effects. Multivariate dispersion as a measure for variability of traits (Anderson et al., 2006) was calculated using PERMDISP (Anderson et al., 2008; results are in Supplementary Table S1). The resulting *z*-values can be interpreted on the scale of the originally chosen dissimilarities (Anderson et al., 2008). All multivariate statistics were performed with Primer 6.1.16 (Clarke and Gorley, 2006).

We used logistic regressions with the fixed factors *population* and *fruit set* to determine whether rarity values of the different floral scent characteristics (total scent quantity, semi-quantitative and qualitative scent patterns) are significant predictors of whether the flowers set fruits [R (The R Core Team, 2019), Packages stats version 3.6.1 and vegan 2.5-6]. In addition, we calculated a model that includes the interaction of rarity and population to test whether the impact of rarity on setting fruit is the same in both populations. A *U*-test (two-sample Wilcoxon test) was used, also in R, to test for differences in rarity between the populations.

## Results

### Composition and Variation of the Floral Scent

The floral scent samples of *C. calceolus* in the two investigated populations varied in absolute quantities from 3.2 to 420 ng/min, and included in total 57 compounds (10–48 per sample, Supplementary Table S2). Terpenoids (25 substances), aliphatic (13), and aromatic (10) compounds were the most numerous, completed by one nitrogen-containing and eight unknown substances. Seven substances were found in more than 99% of the samples: 4-oxoisophorone, heptyl acetate, (*Z*)-3-non-enyl acetate, 1-hexanol, geranylacetone, benzaldehyde, and hexyl acetate (Supplementary Table S2).

The three main compounds, linalool, octyl- and decyl acetate, together contributed 76% to the total scent sampled (linalool: 0–69%, on average 24%; octyl acetate: 0–74%, on average 36%; and decyl acetate: 0–33%, on average 11%) and were, according to a SIMPER analysis, most responsible for the similarity (in sum 79%) in relative amounts of compounds among samples. Half of the other substances were found in traces and contributed in sum for just about 1% of the total scent quantity.

**FIGURE 1:**
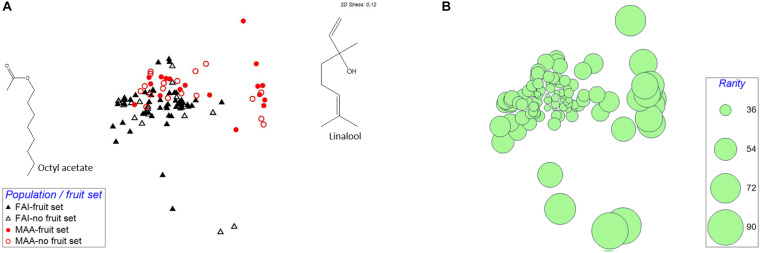
Non-metric multidimensional scaling (NMDS) to visualize semi-quantitative dissimilarities between the individual scent samples taken in the two investigated populations (FAI, Faistenau; MAA, Maria Alm). This ordination is based on pairwise Bray-Curtis similarities. In **(A)**, symbols of scent samples whose flowers have been pollinated are filled, while those of unpollinated flowers are unfilled. **(B)** is the same ordination as **(A)**, here, however, the calculated rarity of the single samples is indicated, with the sizes of the circles being proportional to the rarity of the samples.

The graphic representation of the scent samples indicated at least some variation in relative scent patterns among populations, independent of whether the flowers set a fruit or did not set a fruit (Figure 1A). This was confirmed by permutational multivariate analysis based on relative amounts of scent, which found significant population effects [PERMANOVA: Pseudo-*F*_(__1_,_102__)_ = 16.6; *P* < 0.01], and non-significant effects of fruit set [PERMANOVA: Pseudo-*F*_(__1_,_102__)_ = 1.3; *P* = 0.26] and of the interaction between population and fruit set [PERMANOVA: Pseudo-*F*_(__1_,_102__)_ = 1.2; *P* = 0.29].

### Rarity and Analysis of Negative Frequency-Dependent Pollination

About two thirds of the rarity values, based on relative amounts, in population FAI were in the range between 30 and 40, and the other third spread between 40 and 88. In MAA, 56% of the rarity values were between 30 and 40, the others between 40 and 72 (Figure 1B and Table 1). The graphical representation of rarity in MDS showed that those samples with a larger distance to the majority of samples consistently had larger rarity values than samples with more common scent phenotypes (Figure 1B). The rarity values became consistently smaller the more the data were transformed, i.e., they were largest for untransformed data and smallest for qualitative data (presence/absence-transformation; Table 1). Rarity values differed among populations for relative amounts and their square and fourth root transformations, but not for qualitative data and total quantity of scent (Table 1).

**TABLE 1:** Mean (± *SD*) rarities in various scent properties (non-transformed, square and fourth root transformed relative scent bouquet; presence-absence of compounds; total absolute amounts of scent trapped) of individuals that set fruits and individuals that did not set fruits of two different populations (FAI, Faistenau; MAA, Maria Alm). Rarity values differ between the populations for relative amounts and their square and fourth root transformations, but not for qualitative data and total quantity of scent. Results of logistic regressions (logit) indicate that rarity does not influence fruit set in any of the scent trait analyzed. Since there is no interaction effect with population, influence of rarity on fruit set is not different among the two populations.

Scent	Transformation	Rarity				Population effect on rarity (*U*-Test)	Effect of rarity on fruit set (logit)	Effect of rarity × population on fruit set (logit)
		Population FAI (*n* = 70)		Population MAA (*n* = 36)				
		Flowers with fruit set	Flowers w/o fruit set	Flowers with fruit set	Flowers w/o fruit set			
		Mean ± *SD*	Mean ± *SD*	Mean ± *SD*	Mean ± *SD*	*W*/*p*-value	*z/p*-value	*z/p*-value
Bouquet	Not transformed	38.3 ± 7.7	42.5 ± 15.1	48.4 ± 14.0	43.0 ± 11.4	875/0.01*	−0.3/0.79	1.9/0.06
Bouquet	Square root	34.0 ± 6.9	36.6 ± 12.0	39.4 ± 9.1	36.5 ± 7.7	956/0.04*	−0.3/0.79	1.5/0.13
Bouquet	Fourth root	20.7 ± 6.8	32.6 10.3	33.4 ± 6.0	31.9 ± 5.2	933/0.03*	−0.4/0.66	1.2/0.24
Bouquet	Presence-absence	26.3 ± 7.5	27.9 ± 10.2	26.7 ± 4.0	26.1 ± 3.5	987/0.07	−0.5/0.58	0.7/0.49
Total quantity	Not transformed	153.6 ± 9.7	152.3 ± 10.5	171.7 ± 40.0	164.4 ± 30.0	1017/0.11	0.8/0.44	−0.27/0.78

**FIGURE 2:**
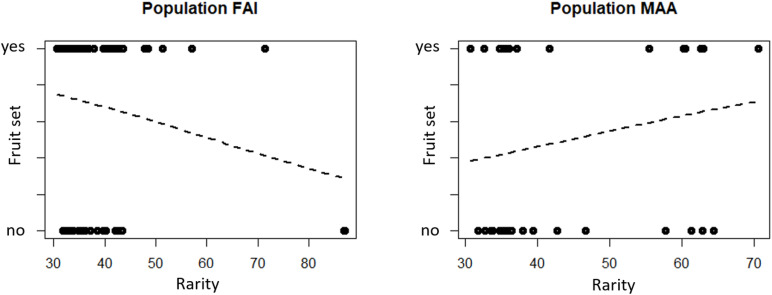
Rarity of scent in flowers with and without fruit set of the two study populations FAI (Faistenau) and MAA (Maria Alm). Rarity values are the mean Bray-Curtis dissimilarities of a sample to all other samples in the population, based on relative amounts of the scent bouquets. Logistic regression results indicate that the rarity of the scent of a flower is not a significant predictors of whether a flower sets a fruit (see also Table 1).

Logistic regressions indicated that rarity is not a predictor of whether a flower sets a fruit, independent of the scent characteristic analyzed (total scent quantity, semi-quantitative and qualitative scent patterns, Figure 2 and Table 1). As indicated by a non-significant interaction effect of *rarity* and *population*, the influence of rarity on fruit set is the same in both populations (Table 1).

## Discussion

We used a recently developed measure to determine uni- and multivariate rarity of floral scent characteristics and found that the rarity of floral scent phenotypes is not a predictor of whether flowers of *Cypripedium calceolus* set fruits. This finding is in line with results of studies on pollinator-mediated negative frequency-dependent selection on floral colors (Pellegrino et al., 2005; Jersáková et al., 2006; Groiß et al., 2017; but see on the contrary Gigord et al., 2001) and on floral scents (frequency of fragrant vs. odorless flowers; Ackerman et al., 1997).

A prerequisite for pollinator-mediated negative frequency-dependent selection, and thus, also for negative frequency-dependent pollination is that pollinators can discriminate among flowers within a population and are capable of learning (Ackerman et al., 1997; Schiestl, 2005). Both conditions seem to be fulfilled in our study system. This is because pollinators of *C. calceolus* have the olfactory capability to detect most of the compounds, among them compounds strongly varying in relative amounts among the samples (Braunschmid et al., 2017) and, although learning abilities in bees have been shown mainly for *Apis melifera* and *Bombus* species (review in Jones and Agrawal, 2017), there is also indication for the learning capability of solitary bees (Amaya-Márquez et al., 2008; Dukas and Real, 1991; Dötterl et al., 2011). Learning implies that pollinators forage (and hence, select flowers) differently depending on their experience. Deceptive plants often benefit from the inexperience of their pollinators, whether they are generally flower-naive or naive regarding the deceptive species. *C. calceolus* is pollinated by more than 30 species of insects (Braunschmid et al., 2017), which emerge and are present at different times. It can therefore be assumed that there is only a limited overlap in phenology of some of these pollinators with the flowering period of *C. calceolus*, making it not possible for them to gain enough experience regarding the different scent phenotypes, and to preferably visit rare ones.

There is evidence that pollinators of deceptive plants only respond to very pronounced unbalancing of the relevant traits. In their study, Jersáková et al. (2006) suspected that negative frequency-dependent selection occurs in natural populations only when color morph frequencies are strongly different. They had no signals for negative frequency-dependent selection in their investigated 22 populations of *D. sambucina*, except for the most unbalanced one with just 7% for one of the two colors. The Gigord et al. (2001) study gives further indications for this hypothesis: fruit set and pollinia deposition (in contrast to pollinia removal) were actually not different between the two color morphs when the frequencies of one of the morphs were 30, 50, and 70% (Figure 3 in Gigord et al., 2001), but were different at frequencies of 10 and 90%. These results suggest that negative frequency-dependent pollination/selection is more likely to occur when some of the phenotypes are particularly rare. In traits like scent, where there often are no distinct chemotypes, but differences among individuals are on a continuous scale, it can be assumed that negative frequency-dependent pollination only acts in populations with both, strongly pronounced differences in scents among individuals and strongly different frequencies of the phenotypes. It appears that at least the latter condition is met in both of our studied populations, with more than half of the samples having similar scents and small rarity values, and about one third of the samples having different scents and higher rarity values (Figure 1). However, we do not know whether the differences in scent are large enough to be recognized by pollinators and whether they are also behaviorally relevant for them.

The frequency of traits attractive to pollinators is not the only aspect influencing the behavior of pollinators. Factors such as the co-flowering community (Jersáková et al., 2006), the spatial variation in pollinator interactions (Chapurlat et al., 2015), and floral abundance (Sabat and Ackerman, 1996) might have significant influence. In consequence, frequency-dependent pollination/selection might no longer be detectable in the behavior of pollinators, and therefore, have no effect on fruit set.

The term rarity has already been used in some previous pollination studies on frequency-dependent selection, but rather informatively, always for categorical traits, and never for comparison of individuals within a population (e.g., Gigord et al., 2001; Iserbyt et al., 2013; Janif et al., 2014). In order to carry out regression analyses with the factor rarity, groups of individuals with trait ratios of $\frac{1}{5}$, $\frac{2}{5}$, $\frac{3}{5}$, $\frac{4}{5}$ etc. were formed and the ratio values used as rarity values. In artificial plots of $\frac{1}{5}$ red flowers and $\frac{4}{5}$ blue flowers, the red flowers were denoted as rarer. Each treatment (e.g., artificial plot of red and blue-flowered plants) served as a replicate with a mean value of fitness measure for a specific phenotype. Mean values of replicate samples were finally used for statistical analysis to compare the fitness of a specific morph in artificial plots that differed in the frequency of this morph. Our approach exceeds such previous studies from a methodical point of view in three aspects: it allows to study (a) multivariate and (b) continuous traits, and (c) assigns the rarity measure to individual phenotypes within a population. The multivariate aspect of data sets that consist of various continuous (e.g., relative amount of scent compounds) or categorical variables (presence/absence of a specific compound) is dealt with the proven means of resemblance measures (e.g., Bray-Curtis dissimilarity, Sørensen dissimilarity). By then calculating the average dissimilarity/distance of a specific subject to all other subjects in the study population, we obtain a measure of rarity for each subject in a population. We used Bray-Curtis, Sørensen, and Euclidean resemblances, but any other dissimilarity/distance measure can be used.

## Conclusion

We have shown how to calculate rarity of multivariate and continuous floral traits, and how to apply the term *rarity* to individual phenotypes in a population. This approach allowed us to demonstrate that the rarity of a flower’s scent does not explain its pollination success in the deceptive orchid *C. calceolus*. Our approach is not restricted to determine rarity of scent phenotypes, but is also applicable to other univariate and multivariate traits.

## Data Availability Statement

The raw data supporting the conclusions of this article will be made available by the authors, without undue reservation, to any qualified researcher.

## Author Contributions

HB and SD designed the research and obtained funds for the research. HB collected and analyzed the data, and wrote the first draft of the manuscript. SD revised the manuscript. Both authors contributed to the article and approved the submitted version.

## Conflict of Interest

The authors declare that the research was conducted in the absence of any commercial or financial relationships that could be construed as a potential conflict of interest. The handling editor declared a past co-authorship with one of the authors SD.
