# Supplementary material for: Does the Rarity of a Flower’s Scent Phenotype in a Deceptive Orchid Explain Its Pollination Success?
Source: Front Plant Sci. 2020 Dec 16;11:584081. doi: 10.3389/fpls.2020.584081 (PMC7772181; doi:10.3389/fpls.2020.584081)
Supplement: Supplementary file 1 [file Table_1.DOCX]

Supplementary Material

# Multivariate dispersion of scent data

There was quite some variation in the fragrance among flowers, with the multivariate dispersion for non-transformed relative amounts based on Bray-Curtis measures being z = 24.3 and z = 31.2 in populations FAI and MAA, respectively. For transformed data the value of the multivariate dispersion decreases with increasing strength of the transformation, and for the qualitative data set were z = 17.0 and z=15.2 in populations FAI and MAA, respectively (Table S1).

Table S1: Multivariate dispersion as a measure for variability of the floral scent bouquet based on relative amounts (not transformed, square and fourth root transformation) and presence-absence of compounds of the two study populations FAI (Faistenau) and MAA (Maria Alm).

|  | Population FAI (n=70) | Population MAA (n=36) |
| --- | --- | --- |
| Transformation | Dispersion (mean ± SD) | Dispersion (mean ± SD) |
| Not transformed | 24.3 ± 1.6 | 31.2 ± 1.8 |
| Square root | 21.7 ± 1.3 | 24.6 ± 1.3 |
| Fourth root | 19.1 ± 1.1 | 20.2 ± 0.1 |
| Presence-absence | 17.0 ± 1.1 | 15.2 ± 0.8 |
